# Stage‐Dependent Inhibitory Connectivity in Striatal‐Motor Circuit in Huntington's Disease

**DOI:** 10.1002/acn3.70104

**Published:** 2025-06-12

**Authors:** Yinghua Jing, Imis Dogan, Rena Theda Overbeck, Kathrin Reetz, Sandro Romanzetti

**Affiliations:** ^1^ Department of Neurology RWTH Aachen University Aachen Germany; ^2^ JARA‐Brain Institute Molecular Neuroscience and Neuroimaging (INM‐11), Research Centre Jülich and RWTH Aachen University Aachen Germany

**Keywords:** amplitude of low‐frequency fluctuation, dynamic causal model, effective connectivity, Huntington's disease, structure atrophy

## Abstract

**Background:**

Elucidating dysfunctional connectivity patterns among key brain regions in Huntington's disease (HD) underlying progression may have implications for developing treatment and therapeutic evaluation.

**Objective:**

Explore the relationship between abnormal spontaneous resting‐state activity and atrophy in HD‐specific brain regions and clarify effective connectivity changes among them across different stages of HD.

**Methods:**

Amplitude of low‐frequency fluctuation (ALFF) analysis was used to detect abnormal spontaneous neural activity; voxel‐based morphometry analysis was applied to assess atrophy; spectral dynamic causal model (DCM) was conducted to estimate regional effective connectivity between HD participants and healthy controls, as well as between preclinical mutation carriers and symptomatic patients.

**Results:**

Voxel‐wise whole‐brain ALFF analysis identified the bilateral caudate nucleus, putamen, and motor cortex as HD‐specific brain regions. ALFF changes in the caudate nucleus and putamen correlated with their respective volumetric atrophy, whereas ALFF changes in the motor cortex preceded its atrophy in the HD preclinical stage. Subsequently, DCM revealed increased inhibitory connectivity from the bilateral caudate nucleus to the motor cortex in HD participants compared to controls. Moreover, compared to preclinical mutation carriers, symptomatic patients showed decreased inhibitory connectivity from the right putamen to the bilateral caudate nucleus, with nonlinear relationships with motor scores.

**Conclusions:**

Our results indicate that striatal atrophy and hyper‐inhibition of caudate‐motorial connectivity might contribute to the regional function alterations in HD. Furthermore, disruption of inhibitory connectivity in the striatal‐motor circuit may play an important role in the emergence of motor symptoms.

## 1 Introduction

Huntington's disease (HD) is an autosomal dominant neurodegenerative disorder caused by a cytosine‐adenine‐guanine (CAG) repeat expansion in the Huntingtin gene (HTT) [1]. Accumulated toxicity of the mutant HTT (mHTT) protein leads to cellular degeneration of neurons in the brain (especially in the striatum), thereby progressively impairing the motor, cognitive, and psychiatric abilities of patients leading to death [2]. Despite the well‐known etiology of HD, the brain connectivity underlying its motor symptoms remains worth further exploration.

The amplitude of low‐frequency fluctuation (ALFF), defined as the mean amplitude of spontaneous fluctuations in the blood‐oxygen‐level‐dependent (BOLD) signal within a low‐frequency range, is a data‐driven method and does not depend on any prior hypotheses [3]. It allows whole‐brain mapping at the voxel level and provides a localized exploration of abnormal spontaneous resting‐state activity. Three previous functional magnetic resonance imaging (fMRI) studies have applied ALFF analysis to explore altered spontaneous resting‐state activity [4, 5, 6]. However, Liu et al. [4] and Kasper et al. [6] only compared symptomatic HD (Symp‐HD) patients with healthy controls. HD has a long preclinical stage, during which neurodegenerative changes occur even though premanifest HD (Pre‐HD) mutation carriers do not present obvious clinical symptoms. Significant striatum atrophy (especially the caudate nucleus and putamen) was already detected by structural MRI studies decades or earlier before the onset of motor symptoms [7, 8, 9]. Furthermore, many task‐based fMRI studies have shown that Pre‐HD mutation carriers exhibited hyper‐activation of motor and cognitive networks (including prefrontal, parietal, and supplementary motor areas) during memory working [10, 11, 12], reward processing [13], and response inhibition [14], possibly as a compensatory response to the initial neuron loss. Thus, it is equally important to include Pre‐HD mutation carriers in examining abnormalities of spontaneous activity in the HD group. On the other hand, Sarappa et al. [5] and Kasper et al. [6] used the fractional ALFF (fALFF) instead of ALFF. The fALFF is the proportion of low‐frequency fluctuations in the overall detectable frequency range [15] rather than the absolute level of low‐frequency fluctuation. Thus, fALFF may be affected by the total amplitude across all frequencies and might not be ideal for the characterization of specific low‐frequency oscillations. Additionally, given that volumetric atrophy of gray matter (GM) is one of the important pathological progressions in HD [7, 16, 17], it is essential to further clarify the relationship between structural atrophy and spontaneous functional activity in abnormal brain regions localized by ALFF analysis throughout HD progression. Most importantly, there is a gap in research exploring how abnormal information flows within these brain regions and differentiating between excitatory and inhibitory connectivity. These all hinder our full understanding of the core neural interaction from the preclinical to the symptomatic stage.

Therefore, following the localization of HD‐specific brain regions associated with HD clinical hallmarks (especially motor symptoms) by ALFF analysis, the main aim of the present study was to examine the relationship between structural atrophy and spontaneous neural activity in HD‐specific brain regions and further clarify the directionality of regional influences among these regions across HD stages. Specifically, voxel‐based morphometry (VBM) [18] was used to extract and assess the structural atrophy in HD‐specific brain regions. Spectral dynamic causal model (DCM) [19] analysis of resting‐state fMRI was performed to construct a biophysically plausible model that provides information on the strength of causal or directed functional connectivity among HD‐specific brain regions.

## 2 Patients and Methods

The study was approved by the local ethics committee (EK083/15, RWTH Aachen University, Germany), and all participants provided written informed consent according to the Declaration of Helsinki.

### 2.1 Participants

Forty‐one individuals with a confirmed CAG repeat (≥ 39 repeats) expansion in the Huntingtin gene and forty‐four healthy controls were enrolled in this study. HD clinical phenotypes were primarily identified by the diagnostic confidence level on motor signs of the Unified Huntington's Disease Rating Scale (UHDRS) [20]. Participants with a diagnostic confidence score ≤ 2 were considered premanifest, and those with a score of 4 were defined as manifest patients. Individuals with a score of 3 represent a category with diagnostic uncertainty and were not included to reduce heterogeneity in group comparisons. In addition, we used the Total Motor Score (TMS) of UHDRS to further characterize motor symptom severity. Pre‐HD had a TMS < 5, exhibiting no or subtle motor symptoms. Thirteen individuals were diagnosed in the preclinical stage, and 28 patients had HD‐specific clinical symptoms. According to the Huntington's Disease Integrated Staging System (HD‐ISS) [17], Pre‐HD individuals were mainly in stage 1, and Symp‐HD patients were mostly classified into stage 3.

### 2.2 Clinical Assessments

All Huntingtin gene expansion carriers underwent thorough neurological assessments. The Disease Burden Score (DBS) [21] was calculated using the formula (CAG repeats − 35.5) × age to estimate cumulative pathology exposure; TMS of UHDRS [20] was used to capture a comprehensive motor symptom; Total Functional Capacity (TFC) of UHDRS and Independence Scale were chosen to measure functional impairment [20]; and Symbol Digit Modalities test (SDMT) [22], Stroop test [23], and Mini‐Mental State Examination (MMSE) [24] were selected to assess cognitive characteristics. Furthermore, the composite UHDRS (cUHDRS) was calculated from TFC, TMS, SDMT, and Word Reading part of the Stroop Test (SWRT) to multidimensionally capture the clinical features of HD progression (Equation 1) [25].

(1) $$\begin{array}{c} \text{cUHDRS}=\left[\left(\frac{\mathrm{TFC}-10.4}{1.9}\right)-\left(\frac{\mathrm{TMS}-29.7}{14.9}\right)\right.\\ \left.+\left(\frac{\text{SDMT}-28.4}{11.3}\right)+\frac{\text{SWRT}-66.1}{20.1}\right]+10 \end{array}$$

### 2.3 MRI Acquisition

All brain imaging data were acquired on a 3 T PRISMA scanner (Siemens, Siemens Healthineers, Erlangen, Germany) with a 64‐channel head coil at the RWTH Aachen University Hospital. During the scanning procedure, earplugs were prepared for all participants to reduce scanner noise, and cushions were carefully placed to minimize head movements. Detailed information about the sequence parameters is provided in Data S1.

### 2.4 Data Processing

**FIGURE 1:**
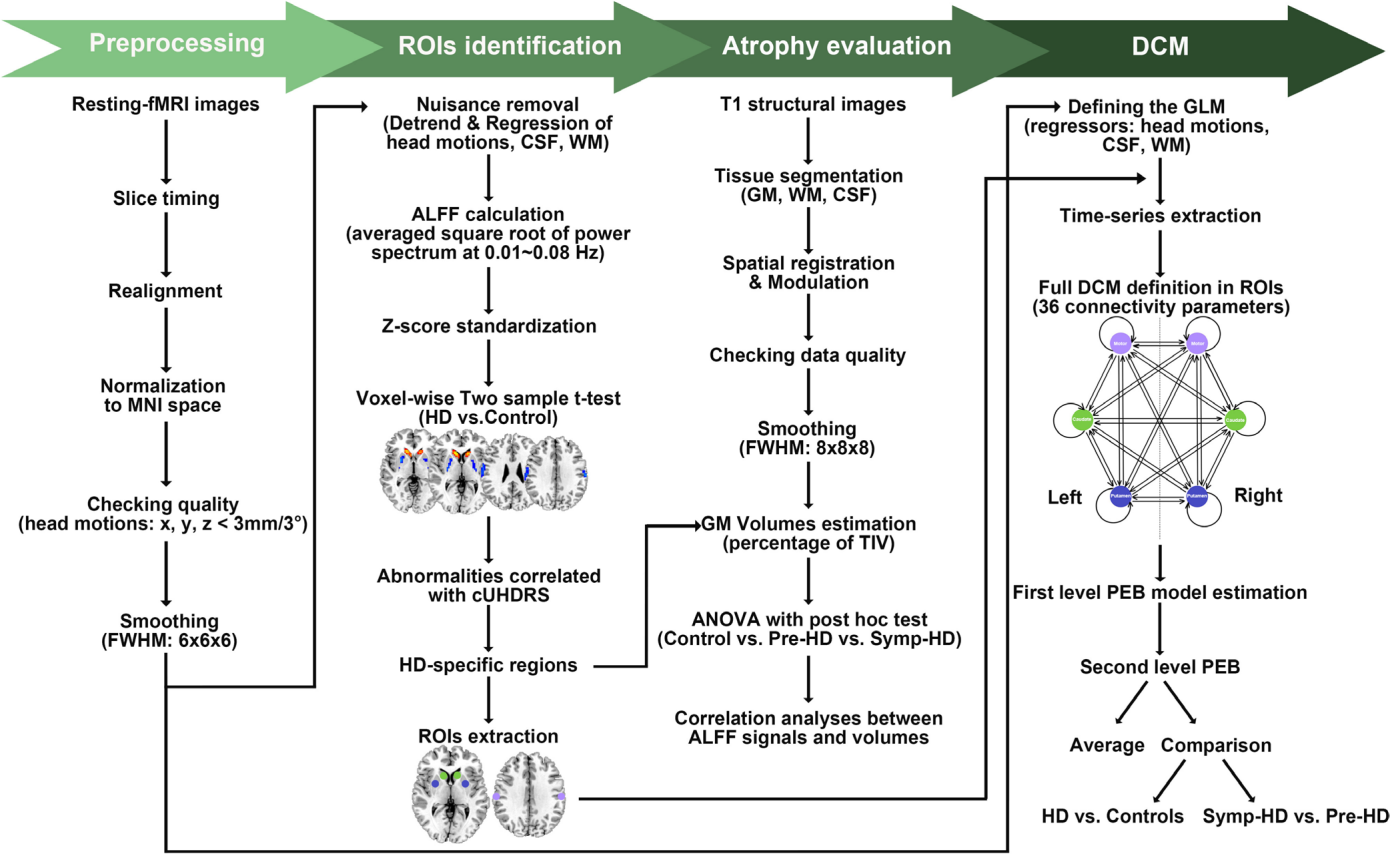
Analytical pipeline of structural and functional data processing. ALFF, Amplitude of Low‐Frequency Fluctuation; ANOVA, Analysis of Variance; CSF, Cerebrospinal Fluid; cUHDRS, composite Unified Huntington's Disease Rating Scale; DCM, Dynamic Causal Modeling; fMRI, functional Magnetic Resonance Imaging; FWHM, Full Width at Half Maximum; GLM, General Linear Model; GM, Gray Matter; HD, Huntington's disease; MNI, Montreal Neurological Institute; PEB, Parametric Empirical Bayes; Pre‐HD, Premanifest Huntington's disease; ROIs, Regions of Interest; Symp‐HD, Symptomatic Huntington's disease; TIV, Total Intracranial Volume; WM, White Matter.

Structural and functional data analyses were performed on MATLAB (version R2019a, MathWorks Inc., Natick, MA, United States) with a combination of CAT12, RESTPlus, and SPM12 toolboxes, following the analytical pipeline as shown in Figure 1. The main steps included: preprocessing of resting‐state fMRI data; whole‐brain ALFF [3] analysis to explore abnormal spontaneous brain activity and to identify the HD‐specific regions of interest (ROIs) as inputs for DCM; VBM [18] to extract GM volumes of HD‐specific brain regions and to assess relationships between volumetric atrophy and abnormal spontaneous neural activity; spectral DCM [19] in the framework of parametric empirical Bayes (PEB) [26] to examine model parameters in a fully connected model and to estimate the directionality of functional influence among those ROIs. For details on data processing, refer to Data S1.

### 2.5 Statistical Analysis

Demographic and clinical characteristics were compared between HD participants and controls as well as between Pre‐HD mutation carriers and Symp‐HD patients, using two‐sample *t*‐tests for continuous variables and Pearson's *χ* ^2^ test for categorical variables. Multiple comparisons of GM volumes and ALFF signals among control, Pre‐HD, and Symp‐HD groups were performed using one‐way analysis of variance (ANOVA) [27] with post hoc contrasts by *t*‐tests. Pearson's correlation analyses were run between ALFF signals and clinical scores, as well as between ALFF signals and GM volumes of HD‐specific brain regions. Statistical significance was set at *p* < 0.05 or Bonferroni corrected *p* < 0.05.

ALFF map statistics of HD participants versus controls were compared using voxel‐wise two‐sample *t*‐tests, with age as an additional covariate. The significance of the resultant T‐map was set at false discovery rates (FDR) [28] corrected *p* < 0.05 and cluster size > 20 voxels.

DCM estimation of within‐group effect and between‐group comparison were computed by PEB analysis using the Bayes rule [29]. Posterior probability (PP) [30] was used to describe the confidence of model parameters. A higher PP value means greater confidence. Thus, the effective connection was considered strongly reliable if PP > 0.99. Further, we performed quadratic regression analyses to assess the potential nonlinear relationships between effective connections and clinical scores. Coefficients of determination (*R* ^2^) > 0.1 and *p* < 0.05 were considered meaningful associations.

## 3 Results

### 3.1 Demographic and Clinical Characteristics

There was no significant gender difference among HD, Pre‐HD, Sym‐HD, and control groups (*χ* ^2^ = 2.755, *p* = 0.252). However, the Pre‐HD group was significantly younger than the Sym‐HD group (*p* = 0.002).

Regarding clinical characteristics in HD subgroups, there was no difference in CAG repeats between the preclinical and symptomatic groups (*p* = 0.079). The Pre‐HD group had lower DBS and TMS, but higher TFC, Independence Score, SDMT (correct), SWRT, and MMSE than the Symp‐HD group (*p* < 0.001). Demographic and clinical details are presented in Table 1.

**TABLE 1:** Demographic and clinical characteristics of the enrolled participants.

	Total HD	Pre‐HD	Symp‐HD	Controls
Number	41	13	28	44
Gender (Male/Female)	17/24	3/10	14/14	20/24
Age (years)	47.88 ± 13.00	38.77 ± 11.23^a^, ^b^	52.11 ± 11.66	46.77 ± 12.01
Handedness (R/L/M)	35/5/1	11/2/0	24/3/1	44/0/0
CAG repeats length	43.56 ± 4.89	42.08 ± 1.98	44.25 ± 5.67	—
DBS	352.49 ± 118.34	249.08 ± 93.23^b^	400.50 ± 96.66	—
UHDRS‐TMS	21.76 ± 19.65	0.54 ± 1.20^b^	31.61 ± 15.94	—
UHDRS‐TFC	9.83 ± 3.32	12.92 ± 0.28^b^	8.39 ± 3.10	—
Independence Score	85.61 ± 14.76	99.23 ± 1.88^b^	79.29 ± 13.79	—
SDMT (correct)	35.41 ± 20.43	60.00 ± 11.55^b^	24.00 ± 11.53	—
SWRT	69.22 ± 27.46	99.38 ± 22.85^b^	55.21 ± 15.61	—
MMSE	26.71 ± 3.97	29.23 ± 1.17^b^	25.54 ± 4.27	—
cUHDRS	11.01 ± 5.68	17.74 ± 2.11^b^	7.88 ± 3.73	—

*Note:* Data are provided as numbers or mean ± standard deviation.

Abbreviations: CAG, cytosine‐adenine‐guanine; cUHDRS, composite UHDRS; DBS, Disease Burden Score; HD, Huntington's disease; L, Left; M, Mixed; MMSE, Mini‐Mental State Examination; Pre‐HD, Premanifest Huntington's disease; R, Right; SDMT, Symbol Digit Modalities Test; SWRT, Word Reading part of the Stroop Test; Symp‐HD, Symptomatic Huntington's disease; TFC, Total Functional Capacity; TMS, Total Motor Score; UHDRS, Unified Huntington's Disease Rating Scale.

^a^ *p* < 0.05 compared to controls.

^b^ *p* < 0.05 compared to Symp‐HD.

### 3.2 Abnormal Spontaneous Neural Activity Related to Huntington's Disease

**FIGURE 2:**
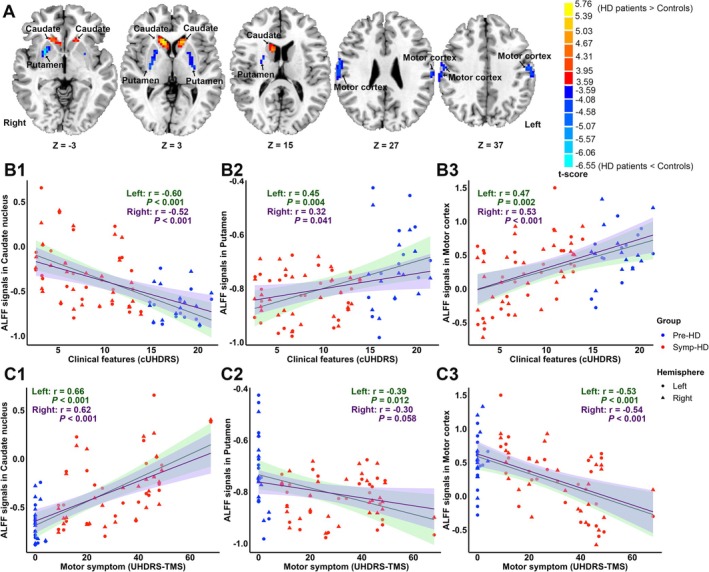
(A) T‐maps with ALFF differences between HD and control groups (FDR corrected *p* < 0.05, two‐tailed test, cluster size > 20 voxels). (B1–B3) Scatterplots and linear regression plots between ALFF signals in HD‐specific brain regions and composite clinical features. (C1–C3) Scatterplots and linear regression plots between ALFF signals in HD‐specific brain regions and motor symptoms. ALFF, Amplitude of Low‐Frequency Fluctuation; cUHDRS, composite Unified Huntington's Disease Rating Scale; HD, Huntington's disease; Pre‐HD, Premanifest Huntington's disease; *r*, correlation coefficient; Symp‐HD, Symptomatic Huntington's disease; TMS, Total Motor Score.

Compared to controls, HD participants showed decreased ALFF signals in the bilateral putamen and motor cortex. In contrast, increased ALFF signals in HD participants were observed in the bilateral caudate nucleus (FDR corrected *p* < 0.05, cluster size > 20 voxels). Detailed differences and spatial maps are provided in Table S1 and Figure 2A.

Furthermore, abnormal ALFF signals in the above‐mentioned brain regions were associated with composite clinical features (Figure 2B1–B3) and motor score (Figure 2C1–C3). Thus, these six brain regions were defined as HD‐specific brain regions for subsequent analyses, and their peak coordinates were used as spherical centers as nodes for constructing the DCM network.

### 3.3 Relationship Between Structure Atrophy and Abnormal Spontaneous Neural Activity

**FIGURE 3:**
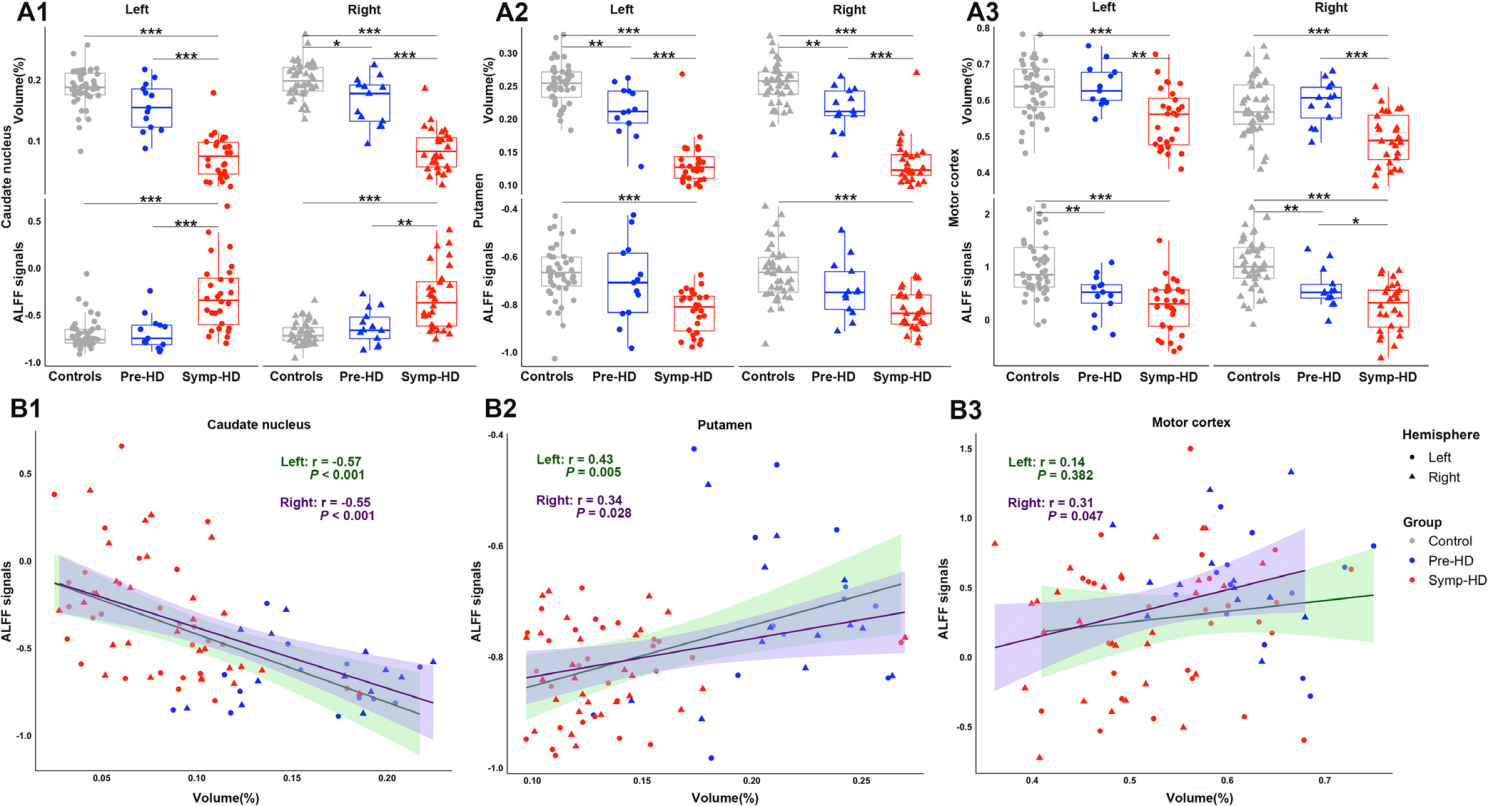
(A1–A3) Alterations of relative volumes and ALFF signals in the caudate nucleus, putamen, and motor cortex among three groups (*p < 0.05, **p < 0.01, ***p < 0.001, Bonferroni correction). (B1–B3) Pearson's correlations between gray matter volumes and ALFF signals in the caudate nucleus, putamen, and motor cortex for patients with Huntington's disease. %, percentage of Total Intracranial Volume; ALFF, Amplitude of Low‐Frequency Fluctuation; HD, Huntington's disease; Pre‐HD, Premanifest Huntington's disease; *r*, correlation coefficient; Symp‐HD, Symptomatic Huntington's disease.

The volumes of bilateral caudate nucleus were reduced in Symp‐HD patients compared to both controls and Pre‐HD subjects (*p* < 0.001), as well as in Pre‐HD subjects compared to controls (*p* = 0.024). However, the Symp‐HD group had higher ALFF signals than both the control and Pre‐HD groups (Symp‐HD vs. Pre‐HD in the right caudate nucleus: *p* = 0.002, other *p* < 0.001). Furthermore, Pearson's correlation analysis in the HD group revealed negative correlations between local ALFF signals and volumes (Figure 3B1).

The volumes of bilateral putamen were decreased in Symp‐HD patients compared to both controls and Pre‐HD subjects (*p* < 0.001), as well as in Pre‐HD subjects compared to controls (left: *p* = 0.007, right: *p* = 0.004). Regarding spontaneous resting‐state activity, only Symp‐HD patients showed lower ALFF signals than controls (*p* < 0.001). Additionally, Pearson's correlation analysis in the HD group showed that the local ALFF signals were positively correlated with volumes (Figure 3B2).

For the motor cortex, the volumes were decreased in Symp‐HD patients compared to both controls and Pre‐HD subjects (Symp‐HD vs. Pre‐HD in the left hemisphere: *p* = 0.001, other *p* < 0.001). Regional ALFF signals were decreased in both Symp‐HD (*p* < 0.001) and Pre‐HD groups (left: *p* = 0.002, right: *p* = 0.004) compared to the control group. Besides, the Symp‐HD group showed lower ALFF signals in the right motor cortex than the Pre‐HD group (*p* = 0.049). Moreover, in the HD group, a positive correlation between ALFF signals and volumes was observed only in the right motor cortex. No significant correlation was found in the left motor cortex (Figure 3B3).

### 3.4 Effective Connectivity Changes Across Stages of Huntington's Disease

**FIGURE 4:**
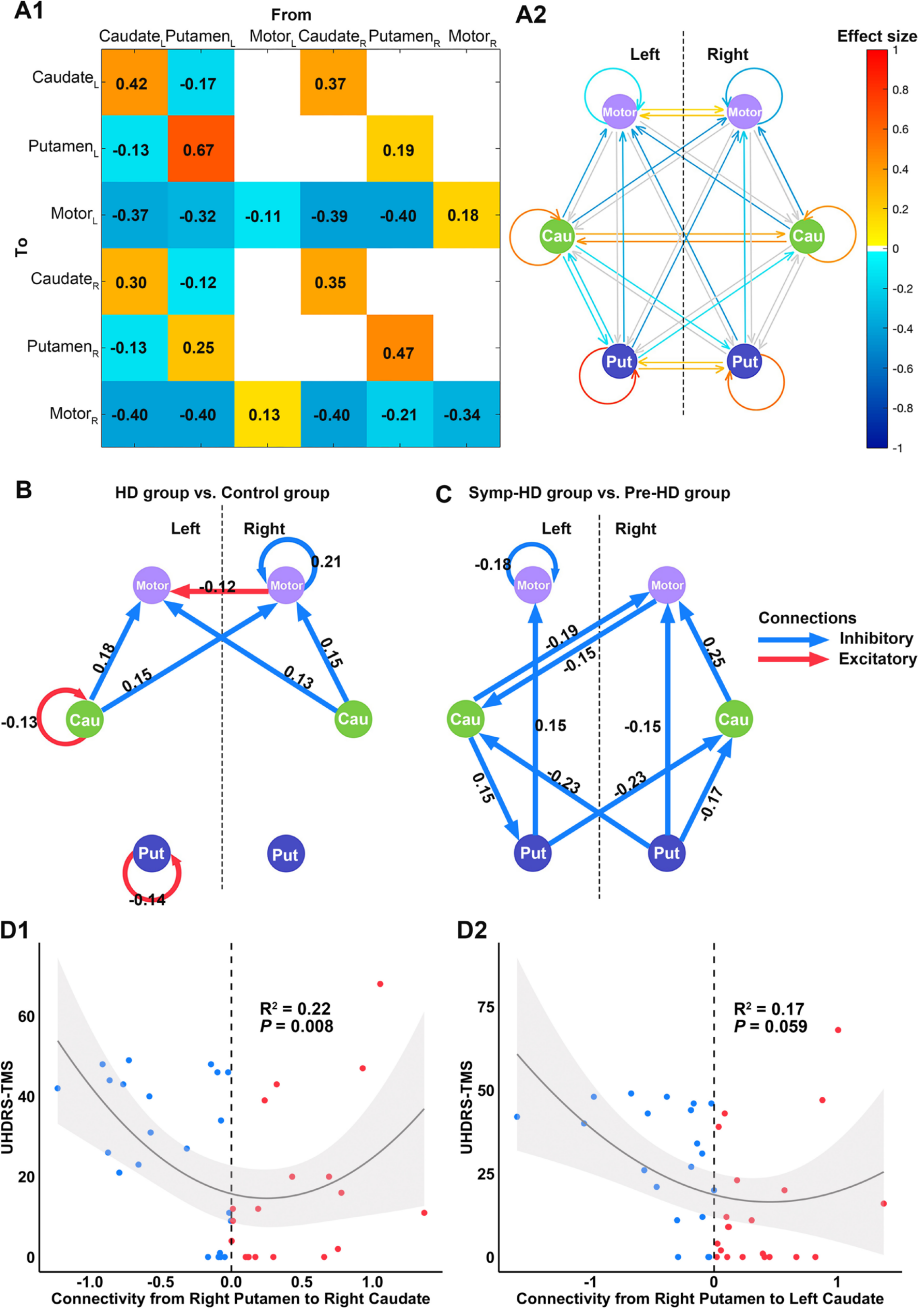
(A1, A2) Within‐group effect of effective connectivity from Bayesian parameter averaging across all participants. (B, C) Results of the Bayesian dynamic model comparison between HD and control groups (B) and between Symp‐HD and Pre‐HD groups (C). (D1, D2) Nonlinear relationships between the specific effective connectivity and motor symptoms. Only significant parameters (Posterior probability > 0.99) are presented. Cau, Caudate nucleus; Pre‐HD, Premanifest Huntington's disease; Put, Putamen; *R* ^2^, coefficient of determination; Symp‐HD, Symptomatic Huntington's disease; TMS, Total Motor Score; UHDRS, Unified Huntington's Disease Rating Scale.

Across all controls and HD participants, the model parameters that best describe the within‐group effect are shown in Figure 4A1,A2, including the connection strengths and connection directions (PPs = 1). Regarding interconnections of the DCM network, there were reciprocal excitatory connections in the caudate nucleus, putamen, and motor cortex to their contralateral homologous brain regions. Additionally, there were inhibitory connections from the left caudate nucleus to the bilateral putamen, from the left putamen to the bilateral caudate nucleus, and from the bilateral caudate nucleus and putamen pointing to the bilateral motor cortex. Furthermore, self‐excitatory connections in the bilateral caudate nucleus and putamen, as well as self‐inhibitory connections in the bilateral motor cortex, were found in the modeled network.

Compared to controls, the inhibitory influences of the bilateral caudate nucleus onto the bilateral motor cortex and self‐inhibitory influence in the right motor cortex were increased in HD participants. Besides, the HD group showed decreased excitatory influence from the right to the left motor cortex and self‐excitatory influences in the left caudate nucleus and putamen (PPs = 1, Figure 4B).

Furthermore, compared to the Pre‐HD group, inhibitory connections in the striatal‐motor circuit were noticeably altered in Symp‐HD patients (Figure 4C). Specifically, the inhibitory influences of the right putamen onto the bilateral caudate nucleus and the ipsilateral motor cortex, from the left putamen to the right caudate nucleus, between the left caudate nucleus and the right motor cortex, as well as self‐inhibitory connectivity within the left motor cortex, were reduced in the Symp‐HD group. On the other hand, Symp‐HD patients showed increased inhibitory connectivity from the right caudate nucleus to the right motor cortex, from the left caudate nucleus to the left putamen and to the left motor cortex (PPs = 1). Notably, inhibitory connections from the right putamen to the bilateral caudate nucleus that showed no within‐group effect were the best discriminative parameters of the between‐group effect in HD subgroups. In addition, the effective connectivity from the right putamen to the right caudate showed a U‐shaped relationship with motor score (Figure 4D1, *R* ^2^ = 0.22, *p* = 0.008). Although the quadratic regression between the connectivity from the right putamen to the left caudate and motor score did not reach significance (Figure 4D2, *R* ^2^ = 0.17, *p* = 0.059), it showed a trend, indicating a possible nonlinear association.

## 4 Discussion

Following the definition of the bilateral caudate nucleus, putamen, and motor cortex related to HD clinical features (especially motor symptoms), this study further revealed that the spontaneous resting‐state activity of these brain regions correlated with their respective structural atrophy, showing negative correlations in the bilateral caudate nucleus and positive correlations in the bilateral putamen. Moreover, we found that alterations of spontaneous neural activity in the preclinical stage in the bilateral motor cortex preceded structural atrophy. In addition, we constructed a DCM network based on the above HD‐specific brain regions and quantified effective connectivity changes, indicating hyper‐inhibitory influences from the bilateral caudate nucleus to the motor cortex in the HD group. Subgroup analysis further revealed dysregulation of inhibitory connectivity in the striatal‐motor circuit underlying motor symptom emergence.

We combined Pre‐HD and Symp‐HD subjects as a whole group for ALFF comparison, which differed from previous studies. Our study identified the bilateral caudate nucleus, putamen, and motor cortex with abnormal spontaneous activity correlated to the composite clinical score and motor score as HD‐specific regions. Besides, we conducted subgroup ALFF analyses comparing the Pre‐HD and Symp‐HD groups with controls, respectively. The subgroup results (shown as Figure S1) were aligned with previous studies [31, 32, 33, 34] and indicated that functional activity in the above‐identified brain regions was associated with disease progression. These findings supported our definition of the bilateral caudate nucleus, putamen, and motor cortex as the HD‐specific brain regions for subsequent analyses.

As shown in postmortem examination of HD brains, loss of medium spiny neurons (MSNs) is the definite pathological feature of HD, beginning in the superior putamen and superior and medial caudate nucleus and progressing toward the base of the putamen and the base and medial of the caudate nucleus [35, 36, 37]. It has been suggested that putamen atrophy occurs earlier and more severely than in other brain regions [17, 38, 39]. Unsurprisingly, our structural measurements showed that volumes of the putamen and caudate nucleus were decreased in HD participants compared to controls, with bilateral putamen atrophy a little more obvious at the preclinical stage. Alterations of the spontaneous resting‐state neural activity in the two regions were not evident until the symptomatic stage, increased in the caudate nucleus but decreased in the putamen. Although the opposite ALFF of the caudate nucleus and putamen may seem at first a counterintuitive finding, in fact, it reflects the profound and complex neuropathological mechanisms of HD and emphasizes the heterogeneity of these key regions in neural degeneration and compensation in different stages. In the preclinical stage, enhanced long‐range functional network connectivity (such as activation of the frontoparietal networks) maintains the level of behavior [10, 11], so the Pre‐HD participants showed no clinical manifestations and no significant change in ALFF compared to controls despite volumetric atrophy. In the late stage of HD, the number of neurons in the striatum is progressively reduced, and synaptic functions suffer severe disruptions. The function may be extremely scaffolded by structural integrity; thus, the local spontaneous neural activity of putamen decreases with structure loss, showing decreased ALFF in Symp‐HD patients. However, the caudate nucleus could over‐activate remaining neurons and enhance synaptic activity in low‐frequency as a compensatory effect for neuron loss in the preclinical (HD‐ISS 1) to mid‐symptomatic (HD‐ISS 3) stage [10, 40]. It is worth mentioning that Kasper et al. reported a reduced fALFF and increased local synchronization in the caudate nucleus, which was absent after correction for volumetric atrophy [6]. As mentioned above, the compensatory activity of the caudate nucleus would increase ALFF, but the remaining neurons in the whole brain of Symp‐HD patients may increase in high‐frequency activity (e.g., beta and gamma oscillations) as in other neurodegenerations [41, 42, 43], which would suppress the relative proportion of low‐frequency activity (reduced fALFF). On the other hand, increased local synchronization or abnormal hemodynamic alterations within the remaining neurons may also lead to increased low‐frequency oscillatory signals in the caudate nucleus, which may not require excessive energy consumption and thus would not contradict hypometabolism in the caudate nucleus as reported by ^18^F‐fludeoxyglucose positron emission tomography studies [7, 44]. Future studies with multimodal imaging studies (combining fMRI, ^1^H, and ^31^P MR spectroscopic Imaging) could help further clarify the nature of this finding.

The striatal‐cortical circuit plays a critical role in HD. Specifically, the motor cortex works with the striatum to coordinate movement, cognition, and emotion [32, 45]. In line with previous studies, our study showed that structural volumes and neural activities in the motor cortex were decreased in HD mutation carriers [5, 6, 46, 47]. Notably, the reduced spontaneous neural activity precedes the volumetric atrophy in the motor cortex in our Pre‐HD group. The motor cortex could release glutamate‐ the main excitatory neurotransmitter in the human brain‐ to activate MSNs in the striatum through their glutamatergic projections, thereby modulating movement signals. When these projections become damaged, glutamatergic stress may hyper‐activate MSNs, leading to striatal neuronal damage [48, 49]. Besides, striatal neuron loss due to glutamatergic stress or inadequate trophic support may promote retrograde degeneration of corticostriatal pyramidal cells [46], showing atrophy of the motor cortex in Symp‐HD patients. On the other hand, the motor cortex can provide brain‐derived neurotrophic factors to support striatal function. Thus, the atrophy of the motor cortex would exacerbate the damage in the striatum. The complex pathologic feedback interactions of the striatal‐cortical loop destine the changes in the structure and function of the motor cortex beyond a simple nonlinear correlation. Moreover, this nonlinear change was biased toward the left, which may be related to a more active metabolism and higher energy demand in the left hemisphere [50].

Further comparison of the DCM network between Symp‐HD and Pre‐HD groups revealed that dysregulated modulation patterns of the striatal‐cortical circuit (especially inhibitory connections) may account for the emergence of HD motor symptoms. Within the striatal‐cortical loop, these cortical projections synapsing with MSNs run two opponent pathways, with direct pathway activity encouraging movement while indirect pathway activity inhibiting or reducing movement [51]. Dysfunction of the indirect pathway is a core mechanism driving the development of motor features in HD, a hypothesis widely referenced [34]. Our findings provide neuroimaging evidence supporting the hypothesis. As MSNs degenerate in the right putamen and disinhibition of indirect pathways occurs, Symp‐HD patients would attenuate inhibitory influences on the ipsilateral motor cortex from the right putamen that fail to control unwanted motor signals input through the striatal‐cortical circuit [35, 52], ultimately resulting in erratic movements. It is more complex in the left hemisphere due to higher vulnerability to regional atrophy [10]. The left motor cortex of Symp‐HD patients would suffer from reduced self‐inhibitory connectivity as an adaptive strategy to neuron loss [53] and be unable to inhibit unwanted motor signals, causing involuntary movements. At the same time, the brain compensatively still enhances the inhibitory influence of the ipsilateral caudate nucleus via the putamen on the motor cortex to modulate the abnormally active motor signs [49]. However, this compensatory mechanism could not be effective and may even lead to further imbalances in motor control in the form of dystonia and rigidity. In addition, our DCM identified that the inhibitory connections from the right putamen to the bilateral caudate nucleus were the best parameters to discriminate the Symp‐HD and Pre‐HD groups, exhibiting nonlinear relationships with motor score. The caudate nucleus and putamen are key structures of the striatum and play key roles in regulatory motor decision‐making [51, 54]. The nonlinear relationship between their connections and motor score reflects complex motor‐regulatory mechanisms within the striatum. We speculate that there might be some dynamic balance of putamen‐caudate connectivity maintaining motor function in the preclinical stage. Weakened inhibitory connectivity from the putamen to the caudate nucleus in Symp‐HD patients indicates a breakdown in the compensatory regulatory mechanisms of the striatum, leading to motor symptoms. This dynamic pattern of effective connectivity emphasizes the importance of stage‐specific interventions in the future.

### 4.1 Limitations

We would draw attention to a few limitations of this study. First, DCM is limited by the necessity of pre‐defined specific regions and only provides a simplified model of the relevant circuits among pre‐defined brain regions. Although it is impossible to model the true extent of anatomical complexity within the striatal‐cortical circuit, we believe this model sufficiently captures the principal dynamics of the functional network or abnormal information flow within the key brain regions. Second, we adopted a cross‐sectional design. A longitudinal study (including full 4 HD‐ISS stages) using multimodal imaging methods would give more details of effective connectivity and metabolism changes among these regions; however, this approach requires several years due to the slow rate of HD progression. Overall, the present study provides only an initial model of HD function, based on cross‐sectional neuroimaging data. Given the complexity and heterogeneity of HD, follow‐up studies involving larger cohorts and longitudinal designs would be essential to validate and extend these findings.

## 5 Conclusion

Taken together, our findings show that striatal atrophy and hyper‐inhibitory influence from the caudate nucleus to the motor cortex might account for the aberrant functional activity in HD. Moreover, the disturbances of inhibitory information flow in the striatal‐motor circuit across different disease stages may characterize the complex disorganization in key brain regions for the emergence of HD motor symptoms. The present study provides neuroimaging evidence supporting the hypothesis of disinhibition of the striatal‐cortical pathway and would give insight into stage‐dependent treatment strategies targeting the striatal‐cortical circuit (especially for noninvasive stimulation therapy).

## Author Contributions

Y.J.: research project: conception and design, organization, execution; statistical analysis: design, execution; manuscript: writing of the first draft, review and critique. I.D.: research project: conception and design, organization; review and critique; manuscript: review and critique. R.T.O.: research project: execution; manuscript: review and critique. K.R. and S.R.: research project: conception and design, organization; statistical analysis: review and critique; manuscript: review and critique.

## Conflicts of Interest

The authors declare no conflicts of interest.

## Supporting information


Data S1.
**Table S1.** Region details with abnormal ALFF signals between controls and patients with Huntington’s disease.
**Figure S1.** (A) T‐maps with ALFF differences between Pre‐HD and control groups (uncorrected *p* < 0.001, cluster size > 5 voxels). (B) T‐maps with ALFF differences between Symp‐HD and control groups (FDR corrected *p* < 0.05, two‐tailed test, cluster size > 20 voxels).
**Figure S2.** Correlation map illustrating Pearson’s correlation coefficients between the ALFF signal in each HD‐specific brain region and clinical variables.
**Figure S3.** (A) Scatterplots and linear regression plots between ALFF signals in HD‐specific brain regions and composite clinical features in the preclinical (A1–A3) and symptomatic stages (A4–A6). (B) Scatterplots and linear regression plots between ALFF signals in HD‐specific brain regions and motor score in the preclinical (B1–B3) and symptomatic stages (B4–B6).
**Figure S4.** Quadratic regression analyses between effective connections and clinical scores.

## Data Availability

The datasets analyzed during the current study are available from the corresponding author upon reasonable request.
