# Supplementary material for: Stage‐Dependent Inhibitory Connectivity in Striatal‐Motor Circuit in Huntington's Disease
Source: Ann Clin Transl Neurol. 2025 Jun 12;12(8):1628–37. doi: 10.1002/acn3.70104 (PMC12343314; doi:10.1002/acn3.70104)
Supplement: Supplementary file 1 — Data S1. Table S1. Region details with abnormal ALFF signals between controls and patients with Huntington’s disease. Figure S1. (A) T‐maps with ALFF differences between Pre‐HD and control groups (uncorrected p < 0.001, cluster size > 5 voxels). (B) T‐maps with ALFF differences between Symp‐HD and control groups (FDR corrected p < 0.05, two‐tailed test, cluster size > 20 voxels). Figure S2. Correlation map illustrating Pearson’s correlation coefficients between the ALFF signal in each HD‐specific brain region and clinical variables. Figure S3. (A) Scatterplots and linear regression plots between ALFF signals in HD‐specific brain regions and composite clinical features in the preclinical (A1–A3) and symptomatic stages (A4–A6). (B) Scatterplots and linear regression plots between ALFF signals in HD‐specific brain regions and motor score in the preclinical (B1–B3) and symptomatic stages (B4–B6). Figure S4. Quadratic regression analyses between effective connections and clinical scores. [file ACN3-12-1628-s001.docx]

**Supplementary Material**

Here we describe in detail:

- MRI Acquisition Parameters.
- Details on Data Processing.
- Supplementary Table 1. Region details with abnormal ALFF signals between controls and patients with Huntington’s disease.
- Supplementary Figure 1. T-maps with ALFF differences between Pre-HD and control groups, as well as between Symp-HD and control groups.
- Supplementary Figure 2. Correlation map illustrating Pearson’s correlation coefficients between the ALFF signal in each HD-specific brain region and clinical variables.
- Supplementary Figure 3. Scatterplots and linear regression plots between ALFF signals in HD-specific brain regions and composite clinical features and motor score in the preclinical and symptomatic stages.
- Supplementary Figure 4. Quadratic regression analyses between effective connections and clinical scores.

**1 MRI Acquisition Parameters**

T1-weighted structural images were obtained by a 3D magnetization-prepared rapid gradient echo sequence with 208 sagittal slices, isotropic voxel of 0.80×0.8×0.8mm, repetition time of 2400 ms, echo time of 2.36 ms, inversion time of 1000 ms, flip angle of 8°, field of view of 288×288mm, and acquisition time was 8.32 minutes.

Resting-state functional magnetic resonance imaging (fMRI) imaging data were collected with a T2^*^-sensitive echo planar imaging sequence with interleaved slice acquisition. Each scan consisted of 270 volumes, 36 slices 3.1 mm thick with an axial plane voxel size of 3.1×3.1 mm covering the whole brain, repetition time of 2210 ms, echo time of 30 ms, flip angle of 90°, field of view of 384×384 mm, and acquisition time was 9.95 minutes.

**2 Details on Data Processing**

*2.1 Functional Data Preprocessing*

All resting-state fMRI data were preprocessed using RESTPlus (version 1.24)^1^. The first 10 time points were discarded to allow the magnetization to reach a steady state. Then, functional images were slice-timing corrected to correct slice-dependent delays. Head motion was corrected by registering raw images to the structural image using six parameters (3 translations and 3 rotations). Successively, spatial normalization to the Montreal Neurological Institute (MNI) space was performed using the deformation fields derived from T1 image unified segmentation (resampling voxel size = 3.0×3.0×3.0mm). After checking the realignment and normalization results, all images were smoothed with a 6mm full width at half maximum (FWHM) Gaussian kernel. No subjects were excluded from further analyses due to large motion (translations in any direction > 3.0 mm or rotation > 3°) or normalization failure.

*2.2 Amplitude of Low-Frequency Fluctuation Analysis*

The amplitude of low-frequency fluctuation (ALFF) analysis was also performed in RESTPlus (version 1.24)^1^. To control systematic and physiologic noise effects, preprocessed data were further detrended to remove the linear trend of time course and were regressed out head movement parameters(using Friston 24)^2^, cerebrospinal fluid (CSF), and white matter (WM). Then, the time course of each voxel was converted to the power spectrum by a fast Fourier transform, and the average square root of the power amplitude across 0.01-0.08 Hz was defined as ALFF^3^. Finally, the ALFF of each voxel was standardized using a Z-score transformation.

*2.3 Regions of Interest Identification*

A two-sample *t*-test for global ALFF maps was performed between HD and control groups to explore abnormal spontaneous neural activity, including age as an additional covariate. The significance of the resultant T-map was set at false discovery rates (FDR)^4^ corrected *P* < 0.05 and cluster size > 20 voxels. Automated Anatomical Labelling (AAL) and Brodmann’s Area (BA) were used to label the peak brain regions of the significant cluster.

Subsequently, Pearson’s correlations between abnormal ALFF signals and clinical scores- composite Unified Huntington’s Disease Rating Scale (cUHDRS)^5^- were analyzed at a significant threshold of *P* < 0.05. Those brain regions with abnormal ALFF values related to cUHDRS were identified as HD-specific brain regions. The MNI coordinates of these HD-specific brain regions were used as spherical centers to create spherical regions of interest (ROIs) with an 8 mm radius, which were then identified as nodes for constructing the dynamic causal model (DCM) network.

*2.4 Voxel-Based Morphometry Analysis*

Voxel-based morphometry (VBM)^6^ analysis on structural data was performed to quantify grey matter (GM) of HD-specific brain regions using the CAT12 toolbox ([https://neuro-jena.github.io/cat//](https://neuro-jena.github.io/cat/)) in SPM12 (<https://www.fil.ion.ucl.ac.uk/spm/software/spm12/>) with default parameter settings. First, T1-weighted structural images were segmented into GM, WM, and CSF. Second, segmented GM images were spatially normalized to the MNI-space via the dimensional Diffeomorphic Anatomical Registration Through Exponentiated Lie Algebra algorithm^7^ and modulated to correct the volumes for regional expansion or shrinkage during spatial normalization. Afterward, all images were checked for homogeneity (to ensure data quality) and smoothed using a Gaussian kernel of 8mm FWHM. Finally, the GM volumes of HD-specific brain regions defined in the previous step were extracted and normalized by total intracranial volume (sum of GM, WM, and CSF volumes) to correct for different head sizes.

*2.5 Dynamic Causal Modeling*

DCM could construct a biophysically plausible model that provides information on the strength of casual or directed functional connectivity between specific regions^8^. After pre-processing, resting-state fMRI data were modeled with a general linear model (GLM) in SPM12, accounting for six head motion parameters, WM, and CSF signals as the nuisance regressors. Then, the principal eigenvariate time series of defined ROIs were extracted from the GLM of each participant to represent regional activity.

For this exploratory study, a data-driven and fully connected model was adopted to assess comprehensively overall and interactive influences within defined ROIs. This means that a total of 36 connectivity parameters for each subject would be generated in all 6 identified ROIs, including interconnections to each other and recurrent self-connections (shown in Fig. 1). Then, spectral DCM in the parametric empirical Bayes (PEB) framework^9^ was applied to assess within-group (all controls and HD participants) and between-group effects (HD group versus control group, Symp-HD group versus Pre-HD group). At the first level of analysis, subject-wise parameters were estimated and inverted to find the estimation of DCMs including connection strength and corresponding model evidence. At the second level, a design matrix was created for modeling group average (commonality) and modeling group comparisons (differences), applying empirical shrinkage priors. Finally, the Bayesian model average from 256 possible model configurations of the 6-node network was used to quantify and infer the expectations and certainties of model parameters.

**References**

1. Jia XZ, Wang J, Sun HY, et al. RESTplus: an improved toolkit for resting-state functional magnetic resonance imaging data processing. Sci Bull (Beijing) 2019;64(14):953-954.

2. Friston KJ, Williams S, Howard R, Frackowiak RS, Turner R. Movement-related effects in fMRI time-series. Magn Reson Med 1996;35(3):346-355.

3. Zang YF, He Y, Zhu CZ, et al. Altered baseline brain activity in children with ADHD revealed by resting-state functional MRI. Brain Dev 2007;29(2):83-91.

4. Genovese CR, Lazar NA, Nichols T. Thresholding of statistical maps in functional neuroimaging using the false discovery rate. Neuroimage 2002;15(4):870-878.

5. Estevez-Fraga C, Scahill RI, Durr A, et al. Composite UHDRS Correlates With Progression of Imaging Biomarkers in Huntington's Disease. Mov Disord 2021;36(5):1259-1264.

6. Ashburner J, Friston KJ. Voxel-based morphometry--the methods. Neuroimage 2000;11(6 Pt 1):805-821.

7. Ashburner J. A fast diffeomorphic image registration algorithm. Neuroimage 2007;38(1):95-113.

8. Friston KJ, Kahan J, Biswal B, Razi A. A DCM for resting state fMRI. Neuroimage 2014;94(100):396-407.

9. Friston KJ, Litvak V, Oswal A, et al. Bayesian model reduction and empirical Bayes for group (DCM) studies. Neuroimage 2016;128:413-431.

**Supplementary Table 1.** Region details with abnormal ALFF signals between controls and patients with Huntington’s disease.

| Peak Brain regions (AAL) | BA | Cluster size (voxels) | Peak *t*-score | Peak MNI coordinate (mm) | | |
| --- | --- | --- | --- | --- | --- | --- |
|  |  |  |  | X | Y | Z |
| **HD patients < Controls** | | | | | | |
| Putamen_R | 48 | 75 | -6.5533 | 27 | 6 | -3 |
| Postcentral_R | 4 | 87 | -5.5648 | 63 | -18 | 30 |
| Putamen_L | 48 | 24 | -4.5474 | -27 | -12 | 3 |
| Postcentral_L | 3 | 51 | -5.112 | -63 | -21 | 33 |
| **HD patients > Controls** | | | | | | |
| Caudate_R | 25 | 104 | 5.7558 | 9 | 18 | 6 |
| Caudate_L | 25 | 55 | 5.317 | -15 | 21 | 3 |

*Note:* All results were corrected for multiple comparisons using false discovery rates (FDR) correction, two-tailed test, *P* < 0.05, cluster size > 20 voxels.

*Abbreviations:* ALFF, Amplitude of Low-Frequency Fluctuation; AAL, Automated Anatomical Labelling; BA, Brodmann’s Area; MNI, Montreal Neurological Institute; HD, Huntington’s disease; R, Right; L, Left.


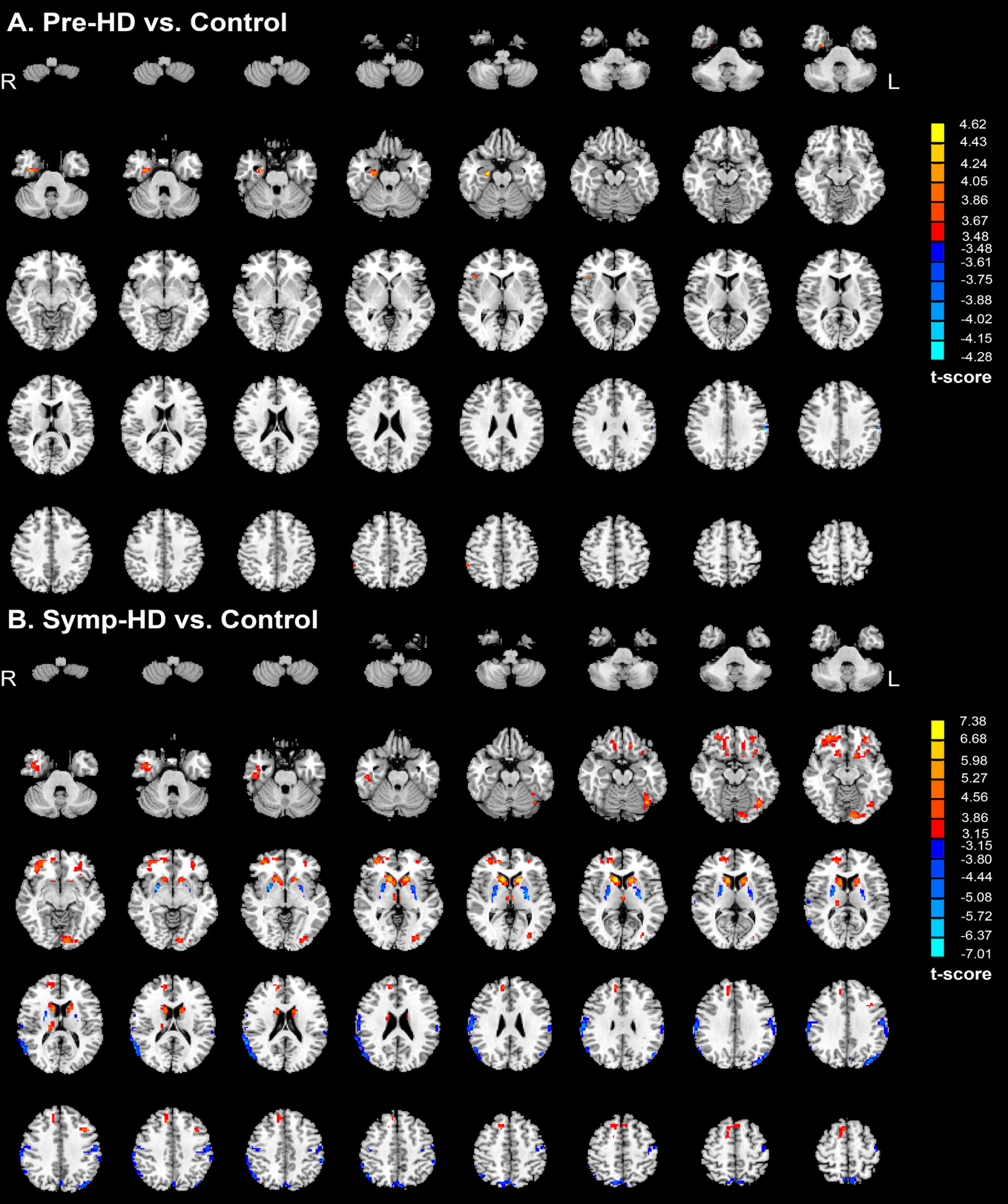


**Supplementary Figure 1.** (A) T-maps with ALFF differences between Pre-HD and control groups (uncorrected P < 0.001, cluster size > 5 voxels). (B) T-maps with ALFF differences between Symp-HD and control groups (FDR corrected P < 0.05, two-tailed test, cluster size > 20 voxels).

Abbreviations: ALFF, Amplitude of Low-Frequency Fluctuation; Pre-HD, Premanifest Huntington's disease; Symp-HD, Symptomatic Huntington’s disease; R, Right; L, Left.


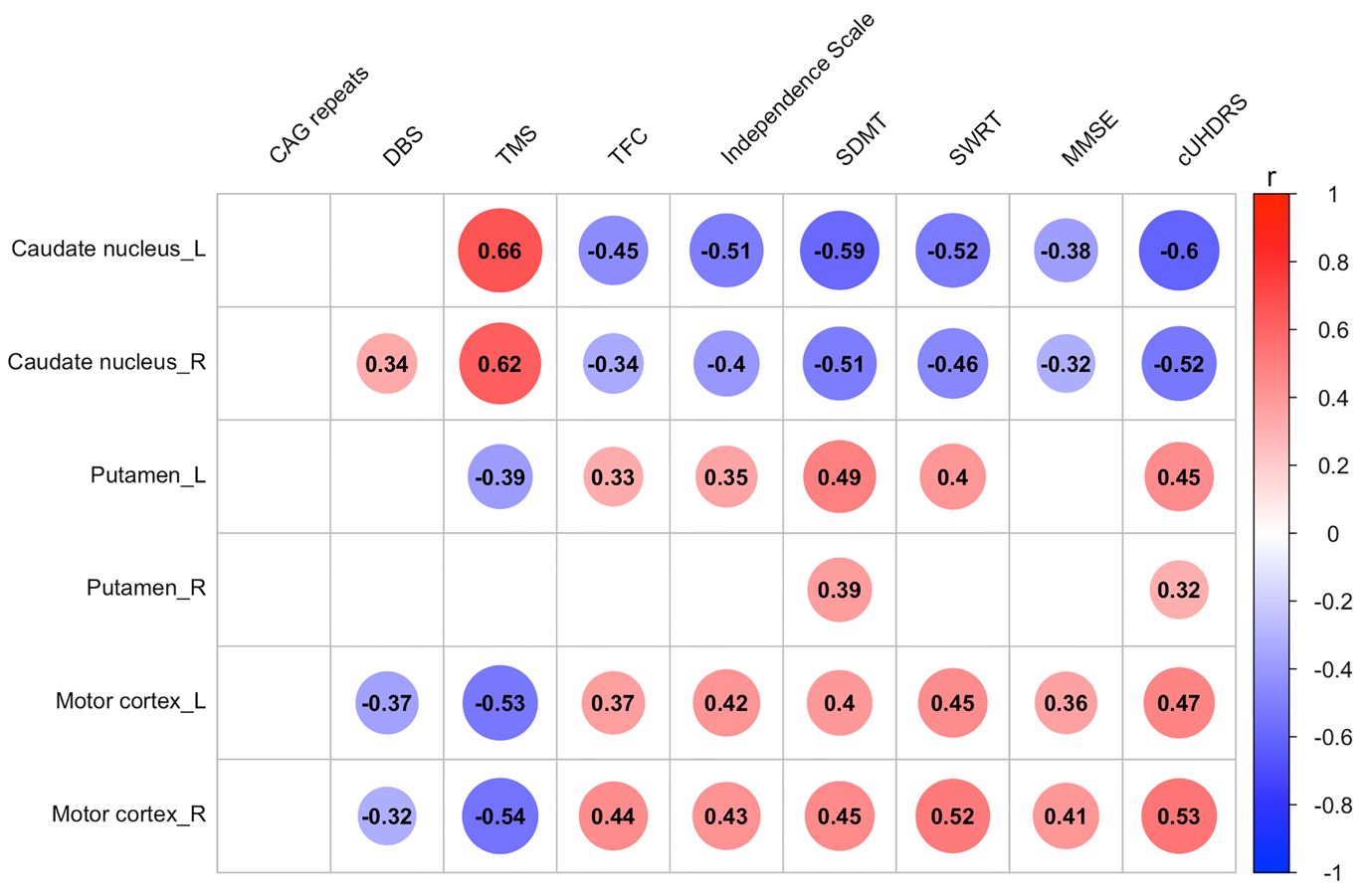


**Supplementary Figure 2.** Correlation map illustrating Pearson’s correlation coefficients between the ALFF signal in each HD-specific brain region and clinical variables.

Only significant correlations (P < 0.05) are presented.

Abbreviations: ALFF, Amplitude of Low-Frequency Fluctuation; HD, Huntington’s disease; CAG, cytosine-adenine-guanine; DBS, Disease Burden Score; UHDRS, Unified Huntington's Disease Rating Scale; TMS, Total Motor Score; TFC, Total Functional Capacity; SDMT, Symbol Digit Modalities Test; SWRT, Word Reading part of the Stroop Test; MMSE, Mini-Mental State Examination; cUHDRS, composite UHDRS; r, correlation coefficient; L, Left; R, Right.


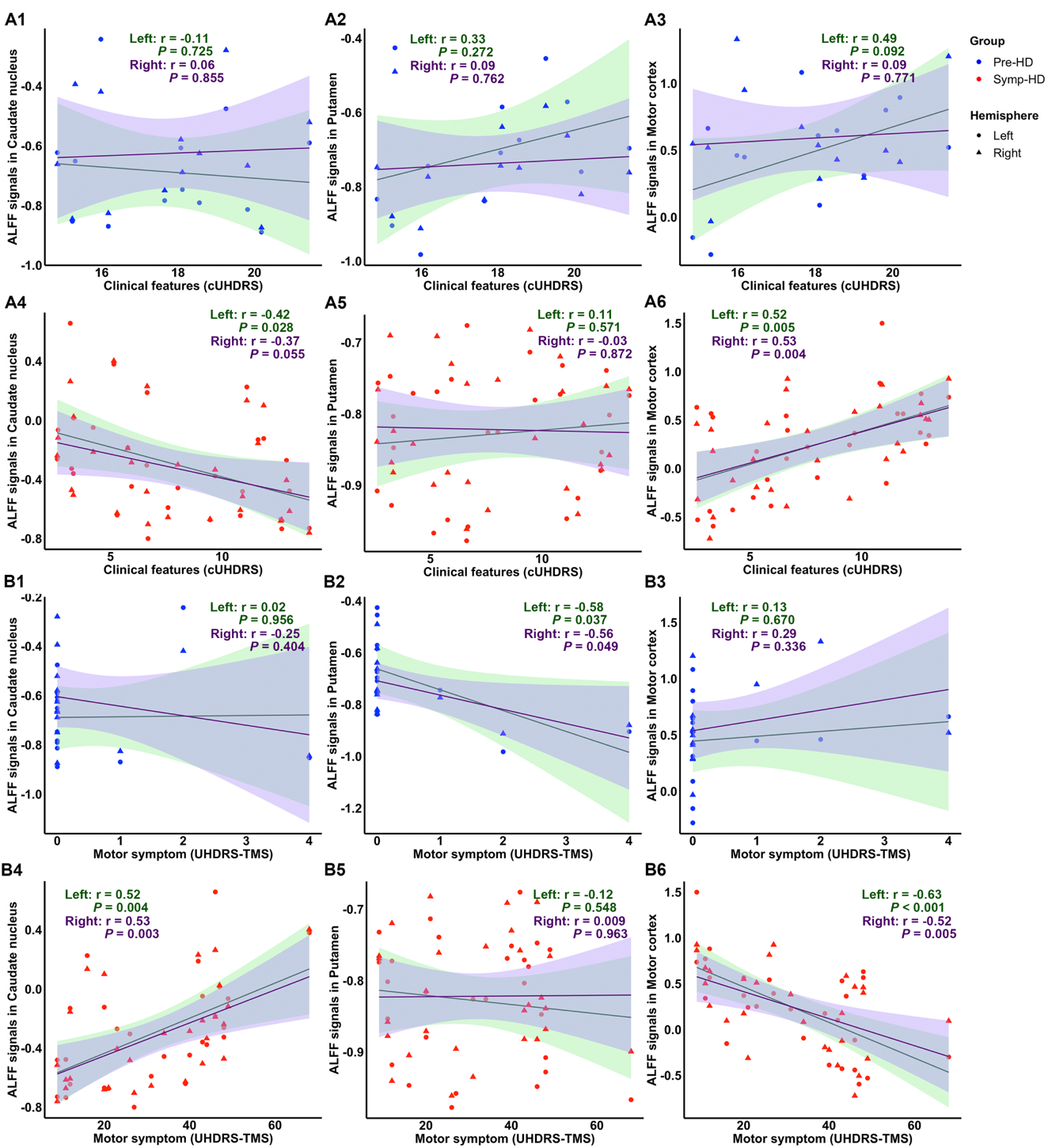


**Supplementary Figure 3.** (A) Scatterplots and linear regression plots between ALFF signals in HD-specific brain regions and composite clinical features in the preclinical (A1-A3) and symptomatic stages (A4-A6). (B) Scatterplots and linear regression plots between ALFF signals in HD-specific brain regions and motor score in the preclinical (B1-B3) and symptomatic stages (B4-B6).

Abbreviations: ALFF, Amplitude of Low-Frequency Fluctuation; cUHDRS, composite Unified Huntington's Disease Rating Scale; TMS, Total Motor Score; r, correlation coefficient; Pre-HD, Premanifest Huntington's disease; Symp-HD, Symptomatic Huntington’s disease.


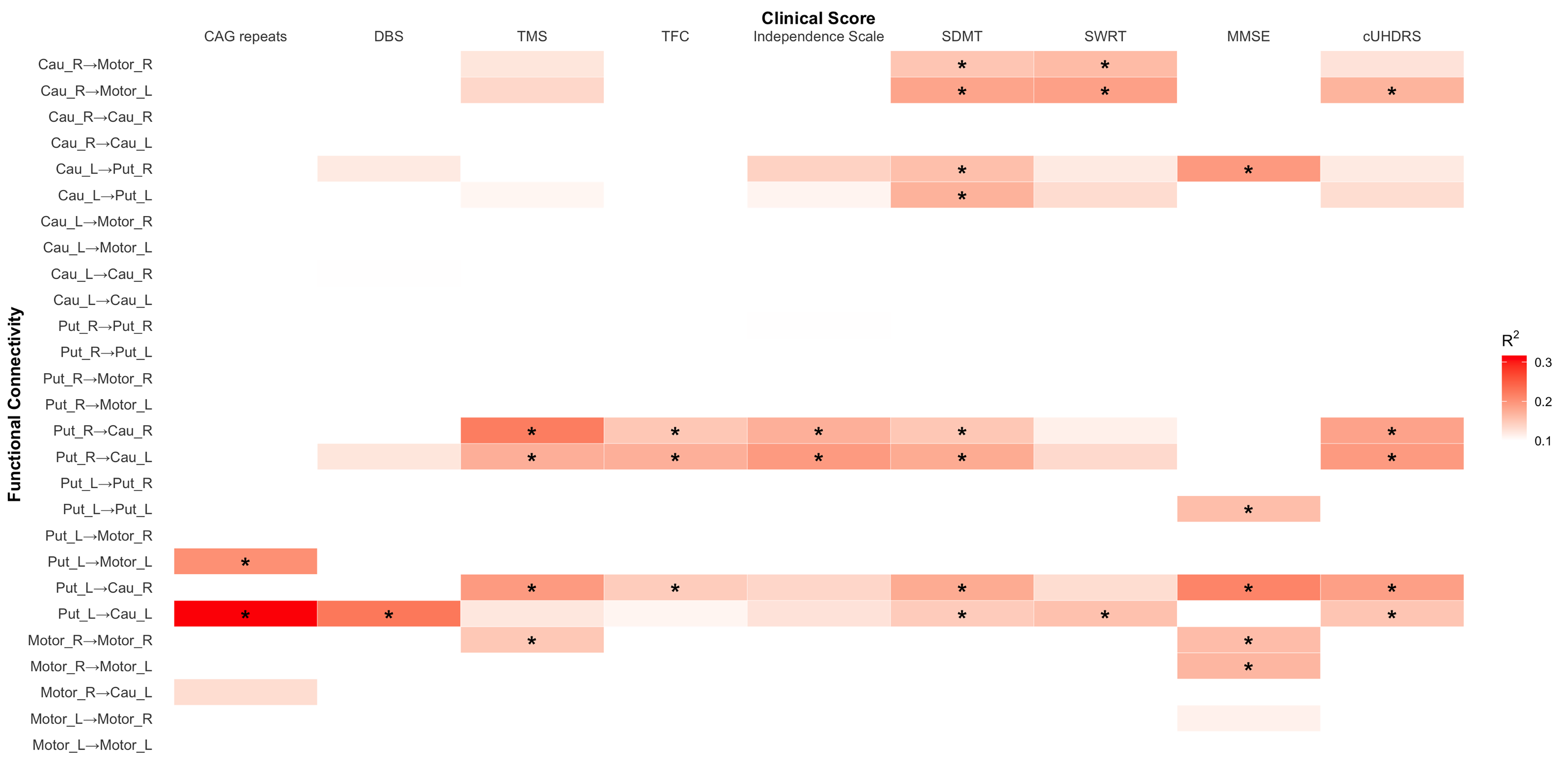


**Supplementary Figure 4.** Quadratic regression analyses between effective connections and clinical scores.

Only models with overall significance (**P* < 0.05) and R² > 0.1 are presented.

Abbreviations: Cau, Caudate nucleus; Put, Putamen; R, Right; L, Left; CAG, cytosine-adenine-guanine; DBS, Disease Burden Score; UHDRS, Unified Huntington's Disease Rating Scale; TMS, Total Motor Score; TFC, Total Functional Capacity; SDMT, Symbol Digit Modalities Test; SWRT, Word Reading part of the Stroop Test; MMSE, Mini-Mental State Examination; cUHDRS, composite UHDRS; R^2^, coefficient of determination.
